# Optimally-Weighted Image-Pose Approach (OWIPA) for Distracted Driver Detection and Classification

**DOI:** 10.3390/s21144837

**Published:** 2021-07-15

**Authors:** Hong Vin Koay, Joon Huang Chuah, Chee-Onn Chow, Yang-Lang Chang, Bhuvendhraa Rudrusamy

**Affiliations:** 1 Department of Electrical Engineering, Faculty of Engineering, University of Malaya, Kuala Lumpur 50603, Malaysia; koayhongvin@gmail.com (H.-V.K.); cochow@um.edu.my (C.-O.C.); 2 Department of Electrical Engineering, National Taipei University of Technology, Taipei 10608, Taiwan; ylchang@ntut.edu.tw; 3 School of Engineering and Physical Sciences, Heriot-Watt University Malaysia, Putrajaya 62200, Malaysia; b.rudrusamy@hw.ac.uk

**Keywords:** optimally-weighted image-pose approach (OWIPA), convolutional neural network (CNN), deep learning, pose estimation, distraction detection, distraction classification, intellegent transport system (ITS)

## Abstract

Distracted driving is the prime factor of motor vehicle accidents. Current studies on distraction detection focus on improving distraction detection performance through various techniques, including convolutional neural networks (CNNs) and recurrent neural networks (RNNs). However, the research on detection of distracted drivers through pose estimation is scarce. This work introduces an ensemble of ResNets, which is named Optimally-weighted Image-Pose Approach (OWIPA), to classify the distraction through original and pose estimation images. The pose estimation images are generated from HRNet and ResNet. We use ResNet101 and ResNet50 to classify the original images and the pose estimation images, respectively. An optimum weight is determined through grid search method, and the predictions from both models are weighted through this parameter. The experimental results show that our proposed approach achieves 94.28% accuracy on AUC Distracted Driver Dataset.

## 1. Introduction

Sustainable transportation systems are vital elements of sustainable cities, and they are aligned with Goal 3 (good health and well-being), Goal 9 (industry, innovation, and infrastructure) and Goal 11 (sustainable cities and communities) of Sustainable Development Goals (SDGs). One of the major factors that influences the transportation system is traffic safety. In United States, about eight people were killed per day in traffic collisions involving distracted drivers [1]. World Health Organization (WHO) also estimates that the number of road traffic deaths will reach 1.35 million in 2020 [2].

Distracted driving is one of the main leading causes of fatal traffic accidents. Actions carried out while driving, such as drinking, turning on the radio, and using a cellphone, could cause a fatal accident. Driving requires a driver’s full attention to safely control the vehicle and respond to events happening on the road. It is a skill that involves constant yet complex coordination between mind and body. A distraction is present when there are events preventing drivers from fully focusing on the driving task. Driver distraction can be categorized into three categories: visual (eyes off the road), manual (hands off the wheel), and cognitive (mind off the task) [3].

Many research efforts aimed to decrease the number of road fatalities while producing a better Intelligent Transportation System (ITS). With the rapid advancement in computer vision and deep learning, many state-of-the-art object detection models have delivered near real-time and accurate results. However, the main issue which is the tradeoff between computational time and accuracy remains a great challenge. Many research achieved accurate detection but slow inference speed. Moreover, the accuracy of the recognition is highly influenced by the input images. Occlusions, lighting conditions, and clutter issues are the common issues that cause poor detection accuracy. These issues are not well addressed in previous studies. Therefore, most of the suggested algorithms or models only work in the daytime but not at night.

While recent research focused on developing specialized convolutional neural network (CNN) models and even using recurrent neural networks (RNNs), we would like to work in the opposite direction using a simple model to outperform them. This study uses only ResNet [4] to perform transfer learning. Many research works [5,6,7] have used the same dataset and have demonstrated the power of RNNs. RNNs are accurate and able to capture spectral features of an image but come with high computation costs. We hope to use the simple state-of-the-art (SOTA) model and evaluate our proposed fine-tuning method to classify the distraction. Moreover, we propose an ensemble of the CNN models that classifies the distractions through pose estimation and original images.

This study aims to detect driver distraction by considering the spatial information based on two modalities of image (RGB and pose estimation image). The dataset used in this study is the American University in Cairo (AUC) Distraction Dataset V2 [8,9]. The main contributions we bring in this work are summarized below:

- Using pose estimation (hand and body pose) classification to classify the distraction.
- Propose Optimally-weighted Image-Pose Approach (OWIPA) to classify distraction through original and pose estimation images.
- Using grid-search algorithm to deduce the weight for maximum prediction accuracy.

The rest of the paper is organized as follows: Section 2 reviews the distracted driver datasets and the approaches used by previous research to detect the distractions. Section 3 explains OWIPA and the CNN models used. Section 4 explains the experiment procedures, evaluation metrics, and the dataset used in this work. Our experimental results and analysis are reported in Section 5. Section 6 presents the conclusion and outlooks of future works.

## 2. Literature Review

Driver distraction detection is one of the most active research areas yet challenging machine learning and computer vision tasks. Researchers tend to detect, localize, and track driver’s body parts such as heads, faces, hands, and gazes to detect distraction. These methods are employed as the basic rule of driving requires the driver to have both hands on the steering wheel and eyes on the road. Therefore, heads and hands are considered to be the key objects to detect driver distractions.

### 2.1. Distraction Detection

Distraction can be defined as any activities that take a driver’s attention away from the task of driving. According to the National Highway Traffic Safety Administration (NHTSA), actions such as rolling down a window and using cell phones are considered to be a distraction [1].

In the early studies of driver distraction detection, only cellphone usage is considered. Therefore, the detection of cellphone usage was the main focus, and many methods were proposed. Moreover, these initial studies use traditional machine learning approaches, such as support vector machine (SVM) and deformable part model (DPM) to extract features from the images. For example, Berri et al. [10] proposed a SVM model to locate face and hand location through the driver’s frontal image view. Craye et al. [11] improved the work by including the occluded images using RGB-D data captured by Kinect sensors. They used AdaBoost and Hidden Markov models to extract arm position, face orientation, action units, and gaze estimation to classify five different postures. Artan et al. [12] used SVMs to detect cell phone usage through a near-infrared (NIR) camera located outside the vehicle directed to the vehicle windshield. They used DPM and SVM to localize and classify the facial landmark, respectively. Moreover, another SVM was used to classify if the driver is using a cellphone. Hidden condition random field (HCRF) was proposed by Zhang et al. [13] to extract the feature from the image captured and classify the usage of a cellphone by driver. Without relying on the location or state of face and hand to assume safe driving behavior, Seshardi et al. [14] proposed a supervised descent method to track face landmarks and AdaBoost classifier to identify the cellphone usage through the left and right face regions.

These studies [10,11,12,13,14] used handcrafted feature learning techniques to extract image features manually. Most of these earlier studies only used a small dataset, with less than 2000 images in both testing and training sets, to evaluate the suggested algorithms. These datasets are not highly varied, with a limited subject (drivers) used in collecting the data. The final classified action was also small and mostly focused on cell phone usage.

In late 2014, more research works started to switch to deep learning methods as they are proven to outperform the traditional machine learning methods. As researchers gave more attention to this field, more distraction actions were considered. More datasets were made available with more distraction actions are being taken into account [8,15,16,17].

In 2013, a vision-based hand activity analysis was conducted by Ohn-bar et al. [18] with the University of California San Diego (UCSD) Laboratory of Intelligent and Safe Automobiles (LISA). They segmented the image into three regions: the wheel, gear, and instrument panel (e.g., radio). A classifier for each segment was developed to detect the existence of hands in those areas. The information gathered was then passed into an activity classifier, which guesses the driver’s actual activity. Later, an extension of the study was conducted to include eye cues [19] and included a secondary back view of the driver [20]. In addition to classification, a region-based classification approach was introduced [21]. The presence of hands in predefined regions in the image was detected. Models were trained for each region separately and were then joined using a second-stage classifier. This proposed system can detect three distractions: adjusting the radio, adjusting the mirror, and operating gear. They improved the classification by diving into more regions to detect more actions taken by the driver [22].

The first dataset considering ten actions was available in 2016 through a Kaggle competition by StateFarm [15]. However, this dataset is not available for research purposes outside the competition [23]. In 2017, Abouelnaga et al. [9] created a new dataset with the same ten actions as the StateFarm dataset, named AUC Distracted Driver Dataset (AUC-DDD). They proposed a real-time distracted driver posture classification. Eraqi et al. [8] produced a model which uses a genetically weighted ensemble of CNN to achieve a 90% classification accuracy. At the same time, they proposed to use two NasNet Mobile [24] models, which reduced the number of parameters, and the model was run in the CPU-based system with an accuracy of 84.64%.

Given the rapid improvement and vast interest in the deep learning field, many state-of-the-art deep learning models [4,25,26,27,28,29,30] were proposed. These models outperform the traditional machine learning methods. Kim et al. [31] proposed to use Inception-ResNet [32] and MobileNet [26] to classify posture distraction. It was shown that fine-tuned models outperformed training from scratch, and MobileNet outperformed Inception-ResNet. However, the dataset used was small and had low variation. Similarly, Alotaibi et al. [7] used ResNet [4], hierarchical recurrent neural network (HRNN) [27] and Inception [32] with minor changes to the model to classify driver distraction. They evaluated their proposed model on StateFarm [15] and AUC-DDD [9] dataset. Likewise, Majdi et al. [33] adopted U-Net CNN to capture context around objects and had shown that it outperformed support vector classifiers on AUC-DDD dataset. A weighted ensemble of AlexNet [25], Inception V3 [28], ResNet [4] and VGG-16 [29] was proposed by Eraqi et al. [8]. Five regions were extracted from the AUC-DDD dataset, i.e., raw image, skin segment, face image, hand image, and face and hand image, to train the CNN. The best result is obtained when raw images were used. The predictions from every CNN were combined through a genetic algorithm (GA) and the results were better than independent CNN and majority voting fusion. Multiple research fine-tuned the pre-trained models and achieved decent accuracy in detecting distractions [34,35].

### 2.2. Pose Estimation

Human pose estimation is the process of inferring poses from an image. It predicts human joints’ positions in an image, also known as the localization of human joints. There are two approaches in human pose estimation, which are bottom-up and top-down. In the bottom-up approach, the processing is done from high to low resolutions, while the top-down approach works the other way round. The top-down approach starts with identifying and localizing person instances through a bounding box object detector. Then, it is followed by estimating the pose of a single person. On the other hand, the bottom-up approach starts by localizing identity-free semantic entities, then grouping them into person instances.

Some of the recent state-of-the-art techniques includes DeepPose [36], DeepCut [37] and OpenPose [38]. The pose estimation techniques used in this work are HRNet [39] and ResNet [40]. HRNet [39] maintains the high-resolution representation of the input data and combines it with high- to low-resolution sub-networks in parallel while reducing computational complexity. It is considered to be top-down approach technique, and the network is built for keypoints estimation based on person’s bounding boxes detected by Faster RCNN [41]. In [40], the authors used ResNet to perform human pose estimation. The method used in this network adds a few deconvolutional layers over the last convolution stage in the ResNet architecture. This structure made it very easy to generate heatmaps from deep- and low-resolution images. It is used to estimate the hand pose in this study.

In addition to human pose, hand pose estimation can be performed too. Hand pose estimation involves modeling the human hand, including palm and fingers, and localizing it in an image. It is considered a subtask of human pose estimation. We use two different networks to perform human pose and hand pose estimation. This is because [40] can provide finer details on hand pose while HRNet [39] can produce precise pose estimation.

Several studies suggested to use body pose estimation for driver assistance system [42] and head pose estimation for distraction detection [43,44]. There are multiple studies of using body pose estimation to classify daily activity actions [45] and human movements [46]. However, there is little attention given to use full body pose estimation in classifying distraction actions. In this work, we would like to explore the usage of CNNs in classifying pose estimation image, instead of classification on keypoints. We treat pose estimation image as another type of image modality.

## 3. Optimally-Weighted Image-Pose Approach (OWIPA)

**Figure 1:**
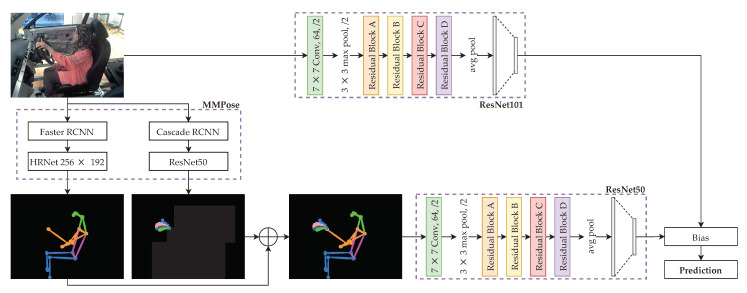
Proposed distraction detection through ensemble of pose estimation classification model and image classification model.

The proposed model is called Optimally-weighted Image-Pose Approach (OWIPA), as illustrated in Figure 1.

First, the original images are pre-processed. All input images are scaled to 360×360 (without cropping) to ensure that all images have the same size. As opposed to the original ResNet’s input size of 224×224, we scale the image to 360×360 in order to capture more features.

In the second stage, the scaled images undergo body and hand pose estimations through two different networks, namely HRNet and ResNet50. After both body and hand pose estimations are completed, the images are combined to produce a complete pose estimation image on black background.

Next, ResNet101 and ResNet50 are trained to classify the original images and pose estimation images, respectively. A weight, ρ is then applied whenever prediction is performed so that one model weight is higher than the other.

Finally, grid search is performed on the trained models to obtain the optimum weights for the highest accuracy.

The first ResNet model is used to classify based on the original images, which are scaled to 360×360. ResNet used in classifying the scaled images is ResNet101, which is pre-trained with Imagenet dataset [47]. ResNet101 is chosen because of the ability to capture the images’ finest details compared to its variants (ResNet18, ResNet34 and ResNet50) and has the best tradeoff between training time and accuracy. The model is trained through transfer learning techniques with the addition of a new head. The new head is located at the final stage of the model, which has ten neurons. This newly-added head is used to classify the distraction based on the predictions from the previous layers.

The second ResNet is used to classify the human pose estimation images. To obtain the human pose estimation images, the original images undergo pose estimation and hand pose estimation. We use two different methods to obtain the human pose estimation, where the body and hand pose estimations are carried out individually.

The body pose estimation is done through HRNet [39]. Before feeding into HRNet, Faster RCNN [41] is used to detect the person to reduce the search space and computational time. Similarly, the original image also undergoes hand pose estimation through ResNet proposed in [40]. Similar to HRNet, before feeding to the pre-trained ResNet on Onehand10k dataset [48], Cascade RCNN [49] is used to detect the hand. Once both the body pose and hand pose estimations are completed, the results are added together to form the full pose images, with a black background. The stitched images are scaled to 360×360, to ensure that results obtained from both ResNets are not biased. The full pose images then undergo classification with pre-trained ResNet50. Both body and hand pose estimation models are pre-trained; therefore, the models are deployed to produce the estimation images without further fine-tuning the model.

**Figure 2:**
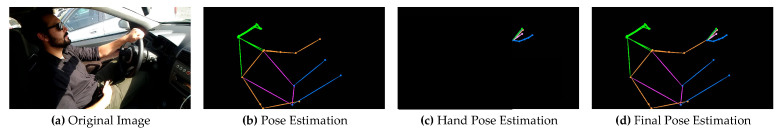
Body and hand pose estimation images.

Instead of using one pose estimation technique to produce the human pose sticks and hand pose sticks, we use two different models to perform the tasks individually and then fuse them, as shown in Figure 2. This is because we want to have finer details on the hand pose estimation since they capture the most information, especially classes that involve hand activities.

Generating pose estimation images are usually time-consuming, and in this work, the average time taken for generating each pose estimation image is around 100ms.

Since both ResNet101 and ResNet50 produce their prediction on each image, a weight, ρ is introduced to the models. The weight is employed such that the prediction of one model weighs more than the other. The weight is determined through a grid-search algorithm with 1000 steps at every validation step while training. The weight is dynamically changed throughout the training, the weight determined by last training epoch is used. Usually, an ensemble of models will use an equal weight, where both predictions will have the same amount of weight. However, we believe that sometimes one of the models will outperform the other in specific classes. The prediction is obtained based on the final weighted prediction.

Table 1 shows the layers in the ResNet101 and ResNet50. The matrix shown in Residual Block layers represents the arrangement of convolutional blocks in the residual block. Please note that we altered the input size and added a head classifier to the pre-trained model.

From Table 1, the last three layers are the newly added head. Since the original ResNet is trained on 1000 classes on ImageNet, the initial fully connected layer is removed and replaced with a new head. The newly added head contains randomly-assigned weights. The pre-trained body is frozen to perform transfer learning efficiently, and the newly added head is trained for several epochs. If the whole network is trained directly without freezing the pre-trained body, the model will be unstable because the newly added head performs badly initially and generates big errors. This will directly impact the pre-trained layers, as the pre-trained weights will be modified while training. Thus, the newly added head is able to train before unfreezing the whole network and training again to ensure the models’ stability and accuracy. After the head undergoes several training epochs, the whole network is unfrozen, and the whole model is trained. These procedures are carried out for both the ResNet50 and ResNet101 models.

The prediction is calculated in Equation (1).

(1) $$\mathrm{Prediction}=\max\left\{\frac{P\times \rho +O\times (1-\rho )}{2}\right\}$$

where ρ is the weight introduced, O is the prediction vector made by ResNet101 on original images and P is the prediction vector made by ResNet50 on full pose images.

## 4. Experiments

### 4.1. Evaluation Metrics

The following metrics are used to evaluate our models.

- Accuracy. It is the proportion of correct predictions among the total number of input samples. Accuracy is considered to be a valid evaluation metric only if the dataset is balanced. High accuracy in an almost equal dataset represents a good model.
- F1 Score. It provides a better measure to predicted result. It is the weighted average of precision and recall, as given in Equation  (2).
  (2) $$F1=2\times \frac{\mathrm{Precision}\times \mathrm{Recall}}{\mathrm{Precision}+\mathrm{Recall}}$$
  F1 score is commonly used when the balance between precision and recall is required. It is a better measure for uneven class distributions, such as a large number of true negative. Therefore, it is preferable for this study since the dataset is uneven.
- Area Under the ROC Curve (AUC). It measures the area underneath the entire ROC curve. It is used to measure the ability of a classifier to distinguish between classes. High AUC represents a perfect model, where it can distinguish between positive and negative classes.
- Cross-entropy loss or negative log-likelihood (NLL) loss. It is used to measure the performance of a classification model, with output of class probability between 0 and 1. This measures the difference between the actual label using the log of the predicted probability. The cross-entropy loss is to produce higher accuracy. The categorical cross-entropy loss is calculated as given in Equation  (3).
  (3) $$\mathrm{Cross}\text{-}\mathrm{entropy}\;\mathrm{Loss}=-\frac{1}{N}\sum_{i=1}^{N}\sum_{j=1}^{M}y_{ij}\cdot \log\left(p_{ij}\right)$$
  where *N* is the number of instances, *M* is the number of classes, $y_{ij}$ is 1 when *i* belongs to class *j* and $p_{ij}$ is the prediction probability of instance *i* belonging to class *j*.

### 4.2. Dataset Description

**Figure 3:**
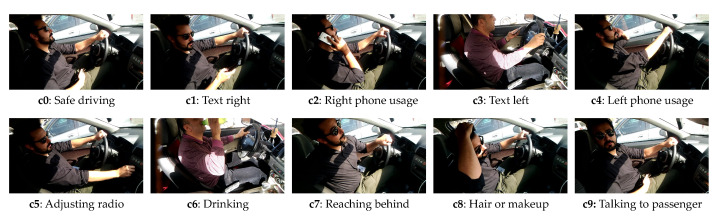
Sample image from each class.

The dataset used in this study is the American University in Cairo Distracted Driver Dataset (AUC-DDD) [8,9]. It is one of the most widely-used driver distraction datasets. The dataset used in this study is the second revision, with more subjects and images. The dataset consists of 44 drivers from seven different countries: Egypt, Germany, USA, Canada, Uganda, Palestine, and Morocco. The videos are recorded on five different cars and at different time of the day to increase the variety of data. The dataset considers 10 distracted actions, as shown in Table 2. The dataset is divided into 80% training and 20% for testing, as per the original split (split by driver) suggested by the author [8,9]. The training dataset is further split into 80% for training and 20% for validation. Please note that the subjects in testing dataset is not found in training and validation dataset. The sample of each class is shown in Figure 3.

### 4.3. Experiment Environment

The ResNet models are trained using Pytorch [50] and *fastai* [51] library while the pose estimations are obtained through *MMPose* [52]. Both model training and pose estimation is carried out on Google Colab, with Tesla T4 GPU. To make sure the result is reproducible, the random seed value is set to 42 throughout the training. To further evaluate OWIPA, we benchmark the model with different variants of ResNets and an ensemble of different combinations of ResNets.

**Figure 4:**
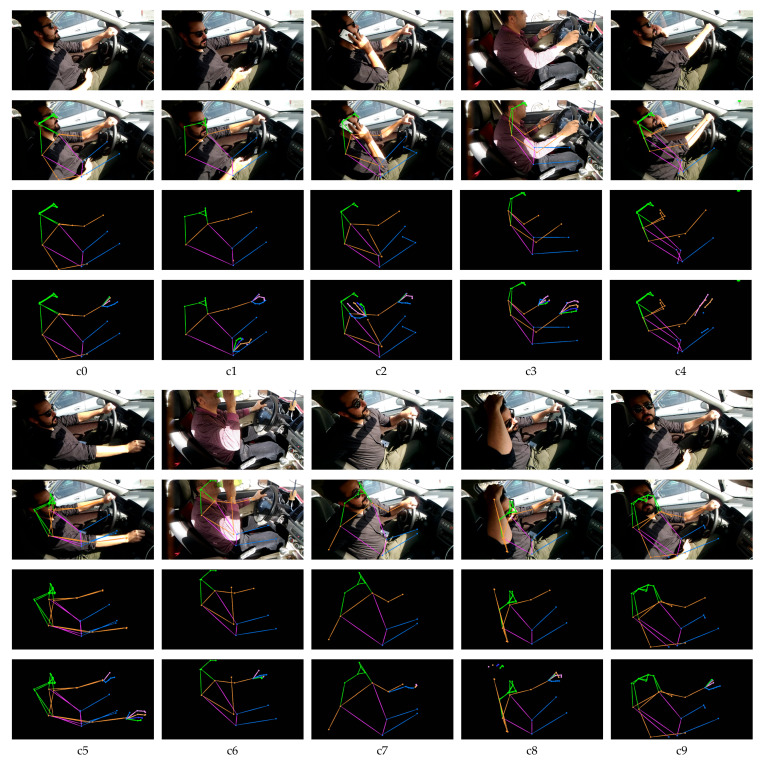
Input images for model training. The first row represents the original image from the dataset. The second row represents the image undergone pose estimation and overlays on top of the original image. The third row represents the pose sticks of the pose estimation. Forth row represents the pose and hand sticks of both pose and hand estimation.

In our experiment, the cross-entropy loss is used as the loss function, and the batch size is all set to 32 to ensure all the experiments can be successfully run on the limited GPU resources. The input images used for training the model is shown in Figure 4.

#### 4.3.1. Pose Estimation

In this work, the pose estimation is done with the pretrained model from *MMPose* library, since they have proven its accuracy. The detection and pose estimation configurations used for body pose estimation are faster_rcnn_r50_fpn_coco and hrnet_w48_coco_256x192, respectively. The detection and pose estimation configurations used for hand pose estimation are cascade_rcnn_x101_64x4d _fpn_1class and res50_onehand10k_256x256, respectively. The pose estimation is performed on every image in the dataset. The performance is acceptable, with prediction loss for both body and hand pose estimation smaller than 0.3. The estimation’s detailed accuracy is not recorded since it does not have ground truth to be verified. As shown in the second row of Figure 4, the pose estimation sticks are mapped onto the driver’s body part, where the head, hand, upper body, and lower body sticks are represented with green, orange, pink, and blue, respectively. The third row is the body pose estimation sticks with the same size’s black background as the original images. The fourth row is the combination of both hand and body pose estimation sticks.

#### 4.3.2. Transfer Learning Procedure

Using a pretrained model for a task different from what it was originally trained for is known as transfer learning. Compared to training a whole new model, transfer learning requires only fine-tuning the parameters by additional training epochs to adapt the dataset. As for transfer learning, the process is carried out in two steps through *fastai* library:

1. The newly added head of the network is trained while preserving the ImageNet [25] weights for the rest of the body. The newly added head is trained for 10 epochs with discriminative learning rate [53] described in Section 4.3.4.
2. The whole network, including body and head of model, is fined-tuned for 20 epochs using discriminative learning rate described in Section 4.3.4.

Generally, when we want to perform transfer learning, we will strip off the head (fully connected layer) and replace with a new one that fit with the number of classes in our dataset. In this case, ImageNet contains 1000 classes, therefore it has 1000 outputs on its fully connected layer, which is not suitable for our dataset. Therefore, we replace it with a randomly-generated weight of fully connected layer (classifier head) with 10 outputs. Since the new layer is of random weights, we will start to fit the layer with our dataset. In this step, the optimizer only updates the weights on this new layer, and the pretrained model body is remained unchanged.

Once the newly-added head is trained with several epochs, the head will be capable of classifying the dataset better. In this work, we train the head for 10 epochs with discriminative learning rate. Higher learning rate is required in this newly-added head, allowing the model to learn faster. Following that, the newly-added head will be stitched back with the other layers from the pretrained model. The whole model will then undergone fine-tuning, updating the intermediate layer parameters to adapt the current dataset. While fine-tuning, a lower learning rate should be used, to allow the model carefully updating the parameters of the other layers. This is because the pretrained model’s body already contain the best feature extraction since it is trained on a large and sparse dataset.

#### 4.3.3. Optimizer

Optimization algorithms are used to update the weights and biases of a model to reduce error. It can be divided into two main categories: a constant learning rate algorithm and an adaptive learning algorithm. The common first-order optimization functions are Stochastic Gradient Decent (SGD), AdaGrad, momentum, RMSProp and Adam. In a recent paper, it is noted that adaptive methods have well-working default parameters, especially Adam [54]. Quoted from [55], “Adam is generally regarded as being fairly robust to the choice of hyperparameters, though the learning rate sometimes needs to be changed from the suggested default”. Therefore, Adam is chosen in this work as the optimizer for all trained models.

#### 4.3.4. Learning Rates

**Figure 5:**
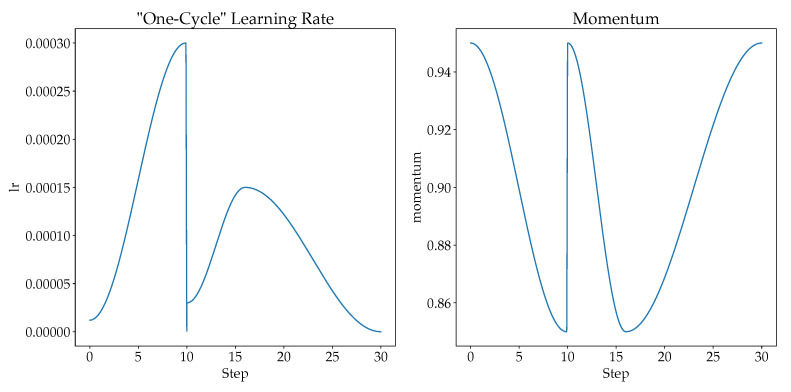
The learning rate and momentum for cosine annealing for our proposed model.

Choosing a suitable learning rate will improve the network’s performance [53], where a small learning rate causes overfitting while large learning rate causes divergence. Therefore, “one-cycle” learning rate policy could be used to solve the learning rate issue. [53] recommends doing one cycle of learning rate of 2 steps of equal length. A lower learning rate is used over the maximum learning rate. Then, the learning rate is tuned from lower learning rate to higher learning rate, and then back to the lower learning rate. When the learning rate is higher in the mid of learning, it serves as a regularization to keep the network from overfitting. The “one-cycle” learning rate policy changes the learning rate after every batch. In this study, we scheduled the learning rates at each epoch based on the cyclical learning rate policy proposed in [53]. The final learning rate and the momentum for cosine annealing are shown in Figure 5.

For the first 10 epochs (training of newly added head classifier), the learning rate is scheduled with a cosine annealing from $4\times 10^{-6}$ to $1\times 10^{-4}$, with momentum for cosine annealing between 0.95 to 0.85.

For the next 20 epochs (unfreezing the whole model), the learning rate is scheduled with a cosine annealing from $1\times 10^{-6}$ to $4\times 10^{-6}$ for first 6 epochs with momentum for cosine annealing between 0.95 to 0.85. The rest of the epochs is scheduled with a cosine annealing from $4\times 10^{-6}$ to $1\times 10^{-6}$ with momentum for cosine annealing between 0.85 to 0.95.

Therefore, it is observed that the learning rate of training the newly added head increases drastically, since rapid training is needed, while the learning rate of fine-tuning increases slowly and decreases slowly too. With “one-cycle” learning rate, the possibility of overfitting is low and models are able to learn quickly and effectively as compared to a fixed learning rate.

## 5. Results and Discussion

We evaluate several ResNet models with different epochs and input image size. The cross-entropy loss (CL), accuracy, F1 Score and individual F1 Score for each class on the AUC-DDD v2 dataset for different ResNet configurations are shown in Table 3. The results are also compared with past studies and shown in Table 4.

Generally, more layers of residual blocks perform better in the classification of original images. From ResNet18 to ResNet101, it is observed that the overall accuracy and F1 score increase. However, the time taken to train the model increases as well. Among ResNet18, ResNet34, ResNet50, and ResNet101, it is observed that ResNet50 has the most tradeoff between training time and accuracy.

The input size of the images affects the accuracy of the model as well. We trained the model with 224×224 and 360×360 images and found that the latter performs better. It is worth noting that the original architecture of ResNet using 224×224 because the category in ImageNet is sparse and has little or no relative relationship between classes. In our case, all images have one person; the difference among them is the hand position and the action happening around the hand and face region. Therefore, larger input images help the model to classify better.

At the same time, more training epochs are needed to train the newly added head classifier. As observed in the “one-cycle” learning curve shown in Figure 4, the learning rate saturated at the 10th epoch as compared to the 5th epoch. As shown in Table 3, all ResNets show a big improvement with 10 training epochs for the newly-added head compared with only 5 training epochs.

As for pose estimation images, it is observed that the time taken to train the model is longer, factoring in the time taken to generate the images. The time taken to produce a pose estimation image in its original size takes about 400ms each with flip testing, and 100ms without flip testing. Pose estimation takes a longer time to process especially when flip testing is enabled. In this work, the images are generated without flip testing. Therefore, all pose estimation images are generated within 30 minutes, and is added to the time taken to train the model. It is also worth noting that the time taken (ATT) reported in Table 4 is calculated with the time taken for model training pipeline to complete. In this case, pose estimation images are generated inside the pipeline before feeding into the network. As for fusion of models, the pipeline is arranged such that one model is trained one after another, and therefore longer time is observed.

The worse performance is observed when only classifying through pose estimation images and does not follow the original image classification trend. Moreover, it is shown that ResNet101 gives worse performance as compared to ResNet50. This means using more layers not only increases the complexity, but also gives poorer detection. This is mainly because the hand region is considerably small compared to the whole pose, even though the noise in the images (the background in the car, including the seat, and steering wheel) is eliminated. Moreover, the driver’s head is now represented with dots, and therefore classes that involve more hand activity will suffer less accuracy. However, it is worth pointing out that pose classification performs better on classes such as adjusting radio because the hand position is relatively far and distinctive. Therefore, we suggest using both the pose classification and original image classification to observe if they bring any improvements.

### 5.1. Selection of Hyperparameter

We performed several iterations of experiments (all sets of training can be found in Appendix A), with varying training hyperparameters. Table 5 summarizes the ablation studies on different hyperparameters used.

From Table 5, we can observed that original image performs better with ResNet101, while pose estimation layer did not perform better when more layers are added. Surprisingly, pose estimation images work wells with all the settings above with ResNet50. Following this idea, we develop several fusion of models with original and pose estimation image.

### 5.2. Fusion of Multiple Models

In the first fusion of ResNets, we combine ResNet50 and ResNet101 models. Both models are trained on original images, so that they perform the best among the others on the same category (with an input size of 360, trained with same epochs). We observe that ResNet50 performs better in class c1, c8, and c9, while ResNet101 performs better in other classes. With this observation, the final predictions are considered through the average of predictions given by both models. With both models’ predictions having equal weights, we observe a slight improvement in overall accuracy and F1 scores. At the same time, it is observed that almost all classes perform better than their individual models.

Following that idea, we fuse models trained with original images and pose estimation images. We use ResNet50 model trained with pose estimation images because it performs the best among the others. In contrast, ResNet50 and ResNet101 models trained with original images since they perform well in some classes over the others. In the first combination of ResNet50 (original images) and ResNet50 (pose estimation images), it is observed that there is a huge improvement in CL, and some classes perform even better, especially class c4 and c5. However, this is not surprising because the model gives close predictions among a few classes in some cases. Averaging them with the model with strong confidence in several classes will then bring huge improvement and thus strong classification. We introduce a weight because pose estimation models perform better in fewer classes than models trained on original images. It is then observed some improvement, even though they might not provide the best prediction for some classes, but they have higher overall accuracy and F1 score.

### 5.3. Performance of OWIPA

**Figure 6:**
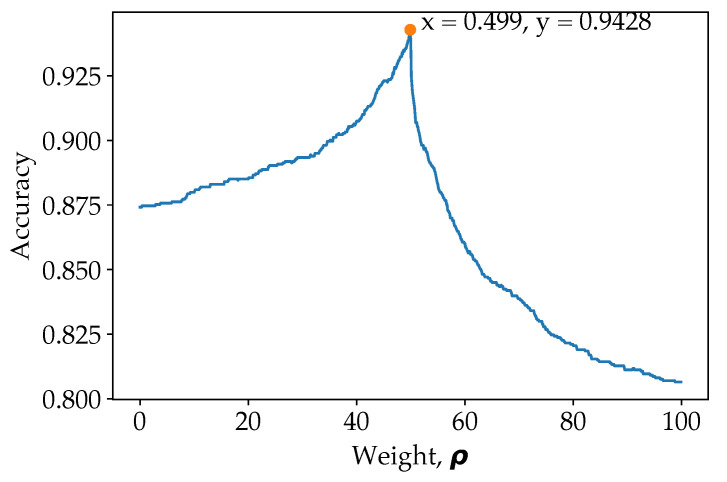
Grid search in determining the optimal weight, ρ.

As for our proposed model (ResNet50 for pose estimation image classification and ResNet101 for original image classification), there is a slight improvement in loss and accuracy after introducing weight. The weight is determined at the last validation step of the training. To visualize the effect of weights, the weight from 0 to 1 with step of 0.001 is shown in Figure 6.

From Figure 6, it is observed that the overall accuracy increases gradually with the weight initially, and when more weight allocated towards the pose estimation over a certain point, the overall classification accuracy reduces drastically. This is because the prediction from model trained on pose estimation images should serve to enhance the prediction from model trained on original images. Predictions from model trained on pose classification images are preliminary used to correct the prediction from model trained on original images for ambiguous cases. For example, when O=[0.03,0.47,0.46,...,0.01] and P=[0.01,0.79,0.18,...,0.03], indicating that the model which classify on original image is confusing between class c1 and c2, while the model which classify on pose estimation image has more confidence on class c2. When applying ρ=0.499, the weightage of class c2 will then be higher, and therefore reducing false prediction. However, when ρ is more than a certain amount, signalling that predictions are preliminary based on pose estimation image model, the F1 score drops drastically. To emphasise once again, the model train on pose estimation images are used to reduce ambiguous or confused class predicted by the model trained on original images. Therefore, it is important to obtain the optimum weight in order to increase the accuracy of the model. Even though the final accuracy improved a little, but it has benefited the prediction of the overall model as a whole, which reduce false predictions. The peak point is when ρ=0.499, where 49.9% of the prediction came from the ResNet50 model (trained on pose estimation images) and 50.1% of the prediction came from the ResNet101 model (trained on original images). This has illustrated the importance of the pose estimation image classification in biasing some of the class, increasing the classification’s accuracy as a whole. The same is also shown in the ResNet50 (original images) and ResNet50 (pose estimation image) model fusion, where more weight is applied to the pose estimation image models.

Furthermore, the time taken for inference from input image to prediction is 850 ms, measured on Tesla T4 GPU. Specifically, the time taken to generate pose estimation image is around 650 ms, while time taken for prediction on both models are around 200 ms.

**Figure 7:**
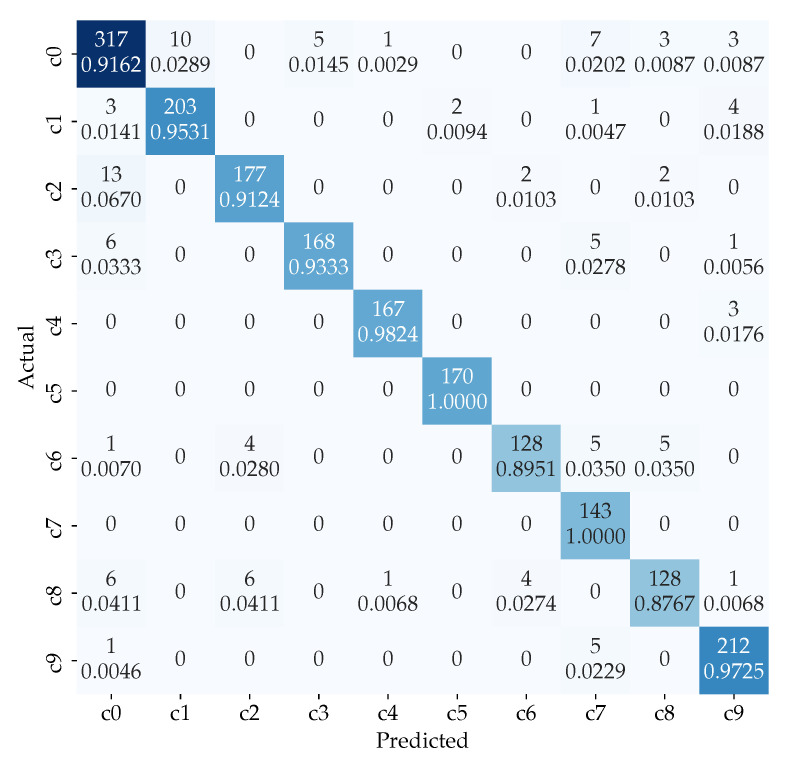
Confusion matrix for the ensemble of ResNet101 model for original dataset classification and ResNet50 model for pose classification with weight.

The confusion matrix of our proposed model is shown in Figure 7. It is observed that the most confused class is class c0 (reaching behind), c2 (right phone usage), c6 (drinking), and c8 (hair or makeup). This is because these class has very similar right-hand activities. For example, as shown in Figure 3, these actions are sometime not visible and is cropped out of frame. Therefore, we use the pose estimation images to reduce the confusion further and therefore uplifting the total accuracy.

**Figure 8:**
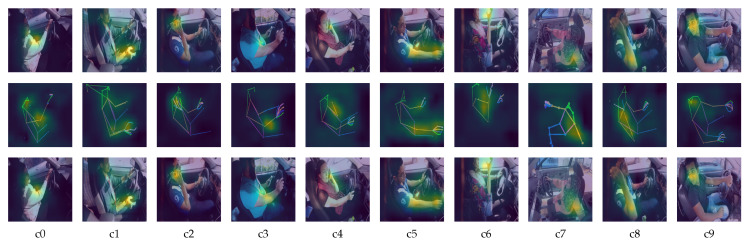
Heatmap of the feature area. First row represents the heatmap generated by ResNet101 on original images. Second row represents the heatmap generated by ResNet50 on pose estimation images. Third row represents the heatmap generated by our proposed method.

To further understand our proposed model, we use the class activation mapping (CAM) technique to highlight the detection area on the model focuses. Figure 8 shows the CAM on ResNet101 (trained on original images), ResNet50 (trained on pose estimation images) and our proposed model. As observed in Figure 8, the ResNet101 which classifies the original images can locate the features quite accurately.

It is shown that both ResNet101 and ResNet50 extract fewer features when executed individually. ResNet101 (trained on original images) tends to focus on the hands and heads, while ResNet50 (trained on pose estimation images) tends to focus solely on the body part. By combining both predictions, the model can focus more area, and therefore producing a more accurate result.

Moreover, since the results are obtained though fusion of two different modality (original image and pose estimation image), the model is less susceptible to lighting condition. There are several pictures in the testing set that contains occlusions and reflection due to sunlight. Through our proposed model, it is observed that there is less wrongly prediction than using ordinary classification model. However, the downside of our proposed model is the computational time, since more steps are taken to produce a single prediction.

Through this work, we found that:

- The usage of "one-cycle" learning rate increases the accuracy and reduces the training loss.
- More epochs are needed to train the newly-added head classifier when performing transfer learning.
- Higher resolution images can increase total accuracy and reduce loss, with minimal increase in total training time.
- The usage of pose estimation images in classifying the class of distraction is useful when coupled with the original image classification model. It is observed that there is about 2% increase in the accuracy as compared to using the original image to perform classification.
- The introduction of weight can increase the accuracy of the model further. Pose estimation images classification should be weighed more to increase the overall classification accuracy.

## 6. Conclusions

Distracted driving is a dangerous act, and one of the prime contributing factors to road traffic accidents. In this work, we propose using pre-trained ResNets to classify 10 classes of distracted driving images inside the vehicle. We use pose estimation techniques to estimate the driver’s pose and then use ResNet to classify the pose images for different distractions. We then propose an ensemble of ResNets, trained on original images and pose estimation images, to classify the distraction. We also introduce a weight to allow the prediction to bias towards one of the models to produce a higher overall accuracy. The proposed model can achieve an accuracy of 94.28% and an F1 score of 94.27%.

For future work suggestion, we suggested that:

- Using keypoint from pose estimation method to classify the action and fuse with the original image classification model.
- Using a dynamic weight for every class. As shown in this work, a fixed weight for overall classification can increase accuracy, but it is observed that some class perform worse than before. Therefore, every class should have its own weight to produce higher accuracy for each class.
- Acquiring video stream of the distracted driving and learning the video’s temporal features, coupled with our proposed model, to produce a better classification.
- Implementing other pre-trained CNNs and applying our proposed techniques to achieve higher accuracy and shorter training time.

## Figures and Tables

**Table 1:** ResNet50 and ResNet101 used in the proposed method, with resized input images.

Layer	Output size	ResNet50	ResNet101
Input	360×360×3	Input image	
Convolution	180×180×64	7×7, 64, stride 2	
Max pool	90×90×64	3×3 max pool, stride 2	
Residual Block	90×90×256	$\left[\begin{array}{c} 1\times 1,64\\ 3\times 3,64\\ 1\times 1,256 \end{array}\right]\times 3$	$\left[\begin{array}{c} 1\times 1,64\\ 3\times 3,64\\ 1\times 1,256 \end{array}\right]\times 3$
Residual Block	45×45×512	$\left[\begin{array}{c} 1\times 1,128\\ 3\times 3,128\\ 1\times 1,512 \end{array}\right]\times 4$	$\left[\begin{array}{c} 1\times 1,128\\ 3\times 3,128\\ 1\times 1,512 \end{array}\right]\times 4$
Residual Block	23×23×1024	$\left[\begin{array}{c} 1\times 1,256\\ 3\times 3,256\\ 1\times 11,024 \end{array}\right]\times 6$	$\left[\begin{array}{c} 1\times 1,256\\ 3\times 3,256\\ 1\times 11,024 \end{array}\right]\times 23$
Residual Block	12×12×2048	$\left[\begin{array}{c} 1\times 1,512\\ 3\times 3,512\\ 1\times 1,2048 \end{array}\right]\times 3$	$\left[\begin{array}{c} 1\times 1,512\\ 3\times 3,512\\ 1\times 1,2048 \end{array}\right]\times 3$
Avg pool	1×1×4096	Average poll, Fully connected	
Flatten	4096	Flatten, BatchNorm	
Dropout	512	Dropout, p=0.25, ReLU, BatchNorm	
Output	10	Dropout, p=0.5, Linear	

**Table 2:** Summary of AUC-DDD.

Class	Description	Training Size (Images)	Validation Size (Images)	Testing Size (Images)
c0	Safe driving	2107	533	346
c1	Text right	1207	298	213
c2	Right phone usage	836	226	194
c3	Text left	762	182	180
c4	Left phone usage	914	236	170
c5	Adjusting radio	745	208	170
c6	Drinking	753	180	143
c7	Reaching behind	716	175	143
c8	Hair or makeup	724	174	146
c9	Talking to passenger	1280	299	218
**Total**		**10,044**	**2511**	**1923**

**Table 3:** The training time (TT), validation cross-entropy loss (CL), accuracy (Acc), F1 Score (F1) and individual F1 Score for each class on AUC-DDD v2 dataset for different configuration of ResNet. Please note that all models are trained with “one-cycle” learning rate, with batch size of 32 and image size of $360^{2}$. Best results are in bold.

Model	ResNet18		ResNet34		ResNet50		ResNet101		ResNet50+ ResNet101	ResNet50 (O)+ ResNet50 (P)		ResNet101 (O)+ ResNet50 (P)	
Data	Ori	Pose	Ori	Pose	Ori	Pose	Ori	Pose	Ori	Ori + Pose		Ori + Pose	
ρ	-	-	-	-	-	-	-	-	-	-	0.498	-	0.499
TT (min) ${}^{a}$	175	210 ${}^{†}$	186	213 ${}^{†}$	209	244 ${}^{†}$	355	388 ${}^{†}$	433 ${}^{‡}$	453 ${}^{‡}$	453 ${}^{‡}$	599 ${}^{‡}$	599 ${}^{‡}$
CL	0.0356	0.0875	0.0284	0.1162	0.0292	0.0613	0.0300	0.0876	0.0296	0.0453	0.0366	0.0457	0.0455
Acc	0.8487	0.8003	0.8544	0.7629	0.8851	0.8066	0.8846	0.7686	0.9012	0.9215	0.9262	0.9376	**0.9428**
F1	0.8444	0.7985	0.8577	0.7693	0.8870	0.8063	0.8874	0.7655	0.9033	0.9210	0.9259	0.9375	**0.9427**
AUC	0.9948	0.9873	0.9936	0.9837	**0.9964**	0.9857	0.9948	0.9821	0.9956	0.9911	0.9932	0.9940	0.9937
c0	0.7010	0.7463	0.8108	0.7458	0.8594	0.8043	0.8650	0.7012	0.8896	**0.9240**	0.9208	0.9231	0.9149
c1	0.9187	0.9057	0.9330	0.7657	0.9067	0.8377	0.8354	0.7100	0.8773	0.9647	**0.9697**	0.9456	0.9531
c2	0.8600	0.7753	0.8496	0.7907	0.9022	0.7650	0.9370	0.8376	**0.9430**	0.9227	0.9391	0.9242	0.9291
c3	0.9422	0.8116	0.8035	0.8042	0.8774	0.8199	0.9003	0.7321	0.9053	0.9330	0.9358	0.9489	**0.9518**
c4	0.8883	0.9471	0.9708	0.9172	0.9426	0.9499	0.9441	0.8951	0.9444	0.9677	0.9676	**0.9853**	**0.9853**
c5	**1**	0.9412	0.9971	0.9226	0.9851	0.9499	**1**	0.9046	**1**	0.9827	0.9913	0.9884	0.9942
c6	0.8947	0.6570	0.9104	0.6240	0.9023	0.6644	0.9254	0.6745	**0.9294**	0.8603	0.8800	0.9084	**0.9242**
c7	0.8624	0.8034	0.7760	0.6272	0.8239	0.7922	0.8299	0.8103	0.8309	0.8800	0.8800	0.9108	**0.9256**
c8	0.7148	0.6300	0.7213	0.6567	0.7569	0.6412	0.7538	0.6145	0.8050	0.8266	0.8375	0.8809	**0.9014**
c9	0.7915	0.7621	0.8238	0.7629	0.9051	0.7931	0.8894	0.8074	0.9024	0.9074	0.9074	0.9522	**0.9593**

${}^{a}$ The training time is calculated on Tesla T4, including the time taken to load data. ${}^{†}$ The training time includes time taken to generate pose estimation images. ${}^{‡}$ The training time for fusion of models are the sum of its individual models training time.

**Table 4:** Comparison of results with previous studies on AUC-DDD v2 dataset (sorted on average accuracy of the model).

Ref	CNN Model	PT	BS	LR ${}^{a}$	Optimizer	Epochs ${}^{b}$	ACL	AA	AF	IT ${}^{c}$
[6]	AlexNet	✓	32	0.0001	Adam	50 (5)	1.024	0.738	0.741	2.61
[8]	GWE-Resnet50 ${}^{†}$	✗	50	0.01	GD	30	0.6615	0.8169	NA	NA
[6]	VGG-19	✓	32	0.0001	Adam	50 (5)	0.531	0.833	0.835	20.46
[7]	HRNN	✓	80	0.001	Adam	30	NA	0.8485	NA	71 ${}^{‡}$
[6]	ResNet50	✓	32	0.0001	Adam	50 (5)	0.442	0.877	0.882	14.26
[6]	InceptionV3	✓	32	0.0001	Adam	50 (5)	0.442	0.884	0.890	22.85
[5]	InceptionV3	✓	NA	NA	NA	NA	0.5723	0.8841	NA	22.85
[6]	InceptionV3-RNN	✓	16	0.0001	Adam	50 (5)	0.418	0.884	0.899	23.42
[7]	ResNet152	✓	80	0.001	Adam	30	NA	0.8852	NA	62 ${}^{‡}$
[6]	Densenet-201	✓	32	0.0001	Adam	50 (5)	0.395	0.890	0.895	46.05
[5]	InceptionV3-LSTM	✓	16	0.0001	Adam	50 (5)	0.4445	0.8982	NA	23.24
[8]	GWE-InceptionV3 ${}^{†}$	✗	50	0.01	GD	30	0.6400	0.9006	NA	NA
[6]	InceptionV3-LSTM	✓	16	0.0001	Adam	50 (5)	0.375	0.902	0.906	23.24
[6]	InceptionV3-GRU	✓	16	0.0001	Adam	50 (5)	0.348	0.903	0.909	23.18
[6]	InceptionV3-BiLSTM	✓	8	0.0001	Adam	50 (5)	0.292	0.917	0.931	23.30
[6]	InceptionV3-BiGRU	✓	8	0.0001	Adam	50 (5)	0.336	0.917	0.922	23.24
[7]	ResNet+HRNN+Inception	✓	80	0.001	Adam	30	NA	0.9236	NA	114 ${}^{‡}$
[5]	InceptionV3-BiLSTM	✗	32	0.0001	Adam	50	0.2793	0.9270	NA	23.30
Ours	ResNet101 (O) + ResNet50 (P) (with weight)	✓	32	1-cycle	Adam	10/20	0.0455	0.9428	0.9427	668.20

**Legends:** PT: Pretrained; BS: Batch Size; LR: Learning Rate; ACL: Average Cross-Entropy Loss; AA: Average Accuracy; AF: Average F1 Score; ATT: Average Training Time (min); IT: Inference Time (sec); NA: Data Not Available. ${}^{†}$ GWE: Genetic weighted ensemble; Original dataset paper. ${}^{‡}$: Collected from original paper. ${}^{a}$: 1-cycle represent the model is trained with one-cycle learning rate policy, as described before. ${}^{b}$: Training head epoch/Training whole network epoch. OR Number of epoch (Patience for early stopping). ${}^{c}$: The inference time is calculated on Tesla T4 for paper without reporting it. All parameters are set according to the original works’ configuration. All models’ batch size is set to 1, iterated over 30 iterations and obtain their average. The code follows the implementation by timm [56] library.

**Table 5:** Ablation studies on hyperparameter setting. F1 score of both models trained on original image and pose estimation image are recorded accordingly. Best results are in bold.

Hyperparamer Setting				Original Image	Pose Estimation Image
Model	Learning Rate	Epochs	Image Size		
ResNet18	0.003	5/10	$224^{2}$	0.3587	0.6543
ResNet18	“one-cycle”	5/10	$224^{2}$	0.7658	0.7300
ResNet18	“one-cycle”	5/10	$360^{2}$	0.8205	0.7480
ResNet101	“one-cycle”	5/10	$360^{2}$	0.7603	0.7510
ResNet101	“one-cycle”	5/20	$360^{2}$	0.8910	0.7545
ResNet50	“one-cycle”	10/20	$360^{2}$	0.8870	**0.8063**
ResNet101	“one-cycle”	10/20	$360^{2}$	**0.8874**	0.7655

**Figure A1:**
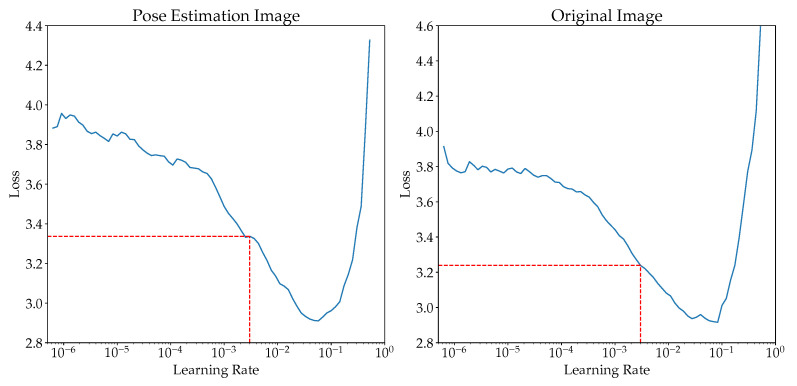
The learning rate search for both datasets.

## Data Availability

Restrictions apply to the availability of these data. Data was obtained from Machine Intelligence group at the American University in Cairo (MI-AUC) and are available from the authors with the permission of MI-AUC.

## Appendix A. Additional Results

All of the experiments conducted during this study is tabulated in Table A1 and Table A2. The experiments are conducted under same environment as described in Section 4.3. The results shown in Table 3 are part of the the following tables.

**Table A1:** F1 Score of different ResNet with and without discriminative learning rate scheduling on original image dataset.

Learning Rate	ResNet18		ResNet34		ResNet50		ResNet101	
	$224^{2}$	$360^{2}$	$224^{2}$	$360^{2}$	$224^{2}$	$360^{2}$	$224^{2}$	$360^{2}$
**Epoch: 5/10**								
lr=0.003	0.3587	0.3033	0.2582	0.3025	0.2465	0.3033	0.3344	0.2477
“one-cycle”	0.7658	0.8205	0.8442	0.8191	0.8115	0.8243	0.8499	0.7603
**Epoch: 5/20**								
lr=0.003	0.4315	0.4355	0.3013	0.2704	0.3356	0.2324	0.2183	0.2255
“one-cycle”	0.8473	0.7801	0.8518	0.8603	0.7801	0.8541	0.7764	0.8910
**Epoch: 10/20**								
lr=0.003	0.4437	0.3598	0.2487	0.2959	0.2069	0.2589	0.2599	0.2673
“one-cycle”	0.7717	0.8444	0.8372	0.8577	0.7682	0.8870	0.8021	0.8874

**Table A2:** F1 Score of different ResNet with and without discriminative learning rate scheduling on pose estimation image dataset.

Learning Rate	ResNet18		ResNet34		ResNet50		ResNet101	
	$224^{2}$	$360^{2}$	$224^{2}$	$360^{2}$	$224^{2}$	$360^{2}$	$224^{2}$	$360^{2}$
**Epoch: 5/10**								
lr=0.003	0.6543	0.7305	0.6676	0.7030	0.6984	0.6713	0.5890	0.5598
“one-cycle”	0.7300	0.7480	0.7660	0.7737	0.7694	0.8012	0.7493	0.7510
**Epoch: 5/20**								
lr=0.003	0.6858	0.6841	0.6809	0.6595	0.6778	0.7053	0.6165	0.6962
“one-cycle”	0.8005	0.7437	0.8007	0.7416	0.7350	0.7257	0.7437	0.7545
**Epoch: 10/20**								
lr=0.003	0.6673	0.6862	0.6681	0.7490	0.6582	0.6307	0.6293	0.5912
“one-cycle”	0.7583	0.7985	0.7624	0.7693	0.7135	0.8063	0.7334	0.7655

### Appendix A.1. Selection of Learning Rate

The value 0.003 is selected based on learning rate search prior to the training. This is achieved using the lr_find function provided by the FastAI library. Figure A1 shows the loss across multiple learning rate.

### Appendix A.2. Effects of Learning Rates

In Table A1 and Table A2, it is observed that “one-cycle” learning rate provides a big improvement than using a fixed learning rate. The effect is more visible especially when the training epoch is small and more features needed to collected from the training data, as shown in Table 1. Improvements as much as 70% in F1 score is observed when training ResNet101 with 5/10 epochs and original images of size $360^{2}$. Therefore, “one-cycle” learning rate is chosen for this study to aid the models in regularization.

### Appendix A.3. Effects of Image Size

Resizing images is one of the important pre-processing step in computer vision task. Usually, we want our models to train faster, therefore smaller images are used. However, selecting an optimal image size would greatly benefited the model. In this work, we found that models trained on $360^{2}$ perform better than that on $224^{2}$, given there is no overfitting or underfitting of model. As shown in Table A1 and Table A2, when the number of epochs are optimal, increasing the image size from $224^{2}$ to $360^{2}$ will benefit the model as much as 10% increase in F1 score.

### Appendix A.4. Effect of Training Epochs

Transfer learning is proven to be the best choice when the targeted dataset is small enough. However, since the models are usually pre-trained with ImageNet dataset, with 1000 classes, therefore the common practice is to remove the last fully-connected layer and add a new layer that suit with the targeted dataset. Since the newly added layer is randomly initialized, it is usually trained with several epochs, while keeping the pretrained layers frozen. Then, the whole model is unfrozen and trained once again. There is no common rule or techniques to set the epochs to train on each process. Therefore, in this work, we start from 5/10 (training the newly added head for 5 epochs, and the whole model for 10 epochs) to 10/20.

As shown in Table A1 and Table A2, it is noticed that even 5/10 performs quite well, when the model does not present any bottleneck. For example, ResNet101 for original images with image size of $360^{2}$ can achieve 0.8497 F1 score, with training time taken less than 70 min. However, most of the time, 5/10 and 5/20 met the bottleneck since more features are to be learnt but the training step is simply not enough, therefore the model did not generalize well.

Through these training processes, it is observed that:

- Usage of “one-cycle” learning rate will help the model to regularize well, even with less training epochs.
- Higher resolution of images will help the model to capture more features. However, more training epochs are needed.
- Selection of training epochs should be balanced between training of newly added head and the whole models. Early stopping could be deployed as well.
